# Tegumentary Leishmaniasis Associated With Immune Reconstitution in an HIV Patient—A Case Report

**DOI:** 10.1111/pim.70082

**Published:** 2026-05-11

**Authors:** Marilia Brasil Xavier, Claudia Maria de Castro Gomes, Rita Catarina Medeiros Sousa, Edna Aoba Yassui Ishikawa, Elza Baía de Brito, Silvia Ferreira Rodrigues Müller, Lucas dos Santos Fontes, João Augusto Gomes de Souza Monteiro de Brito, Larissa dos Santos Alcantara, Carlos Eduardo Pereira Corbett

**Affiliations:** ^1^ Research Laboratory in Tropical Dermatology and Endemic Diseases, Nucleus of Tropical Medicine Federal University of Pará Belém Pará Brazil; ^2^ Infectious Diseases Pathology Laboratory (LIM50), Pathology Department, Medical School São Paulo University São Paulo São Paulo Brazil; ^3^ Medicine School Federal University of Pará Belém Pará Brazil

**Keywords:** coinfection, HIV, immune reconstitution inflammatory syndrome, immunohistochemistry, macrophage activation, mucocutaneous leishmaniasis, Th2 cells

## Abstract

HIV‐associated Immune Reconstitution Inflammatory Syndrome (IRIS) may significantly alter the immunopathological presentation of American Tegumentary Leishmaniasis (ATL), occasionally causing paradoxical clinical exacerbations. We report the long‐term follow‐up of a 39‐year‐old female coinfected with HIV and disseminated mucocutaneous leishmaniasis caused by *Leishmania* (*Viannia*) sp., who experienced severe lesion exacerbation four months after initiating High‐Activity Antiretroviral Therapy (HAART). Despite successful viral suppression and CD4+ T‐cell recovery, she developed aggressive mucocutaneous plaques with nasal septum destruction. Immunohistochemical analysis of a skin biopsy revealed a profile distinct from HIV‐negative ATL controls: classic pro‐inflammatory markers (CD68, iNOS, IL‐6, and IL‐17) were markedly suppressed, while CD163, IL‐10, TGF‐β and IL‐18 were elevated, signalling M2 macrophage activation and paradoxical Th2 polarisation. CD8+ T cells were the most preserved lymphocyte subset, which is consistent with their reported cytotoxic, tissue‐damaging role in mucosal leishmaniasis caused by *L.* (*Viannia*) *braziliensis*. Standard pentavalent antimonial combined with sustained HAART led to complete resolution without recurrence over 16 years. This case illustrates how IRIS may be associated with atypical Th2‐polarised pathology and CD8‐mediated tissue injury in ATL, highlighting the need for awareness of this presentation in coinfected patients from endemic areas.

## 1 Introduction

HIV/leishmaniasis coinfection is an emerging condition, particularly in regions where the two diseases overlap geographically, as in Brazil [1, 2]. Visceral leishmaniasis has been more frequently reported as coinfection, particularly in Mediterranean countries, presenting with atypical manifestations, refractoriness to standard therapy and frequent relapses [3, 4, 5]. Brazil accounts for most registered coinfection cases in South America, predominantly involving American Tegumentary Leishmaniasis (ATL), with *Leishmania* (*Viannia*) *braziliensis* and *Leishmania (Leishmania) amazonensis* as the species of greatest medical importance [2]. ATL presents a broad clinical and immunopathological spectrum, ranging from localised cutaneous leishmaniasis (LCL) to the polar forms, anergic diffuse cutaneous leishmaniasis (ADCL) and mucocutaneous leishmaniasis (MCL). This spectrum is strongly associated with the *Leishmania* species, its virulence and the host's immunogenetic profile [6, 7, 8].

High‐Activity Antiretroviral Therapy (HAART) has transformed the course of HIV disease and, in most coinfected patients, it contributes to leishmaniasis control rather than exacerbation [9, 10]. Paradoxically, however, the rapid recovery of CD4+ T cells and viral suppression may worsen latent or ongoing infections, a phenomenon known as Immune Reconstitution Inflammatory Syndrome (IRIS). IRIS has been described in association with *Mycobacterium avium* , cytomegalovirus, leprosy and, more recently, ATL [9, 11, 12, 13, 14].

This report describes a case of HIV/ATL coinfection associated with IRIS, focusing on clinical evolution, immunopathology and long‐term outcome, in order to contribute to the understanding of coinfection dynamics in the context of immune restoration.

## 2 Materials and Methods

### 2.1 Case Presentation

A 39‐year‐old female patient from Dom Eliseu, Pará state, Brazil, an endemic area for ATL, with a history of untreated cutaneous leishmaniasis in childhood, was followed at the Research Laboratory in Tropical Dermatology and Endemic Diseases of the Federal University of Pará. In October 2007, the patient presented with disseminated MCL. Montenegro skin test was negative and PCR from a cutaneous lesion was positive for *Leishmania* (*Viannia*) sp. HIV‐1 serology (ELISA) was positive, with a CD4^+^ T‐lymphocyte count of 87 cells/mm^3^ and a viral load of 443,905 copies/mL. Pentavalent antimonial therapy was initiated, but adherence was poor. HAART was started in April 2008 (zidovudine 600 mg + lamivudine 300 mg + efavirenz 600 mg), concurrently with a new cycle of pentavalent antimonial; again, adherence was inadequate.

**FIGURE 1:**
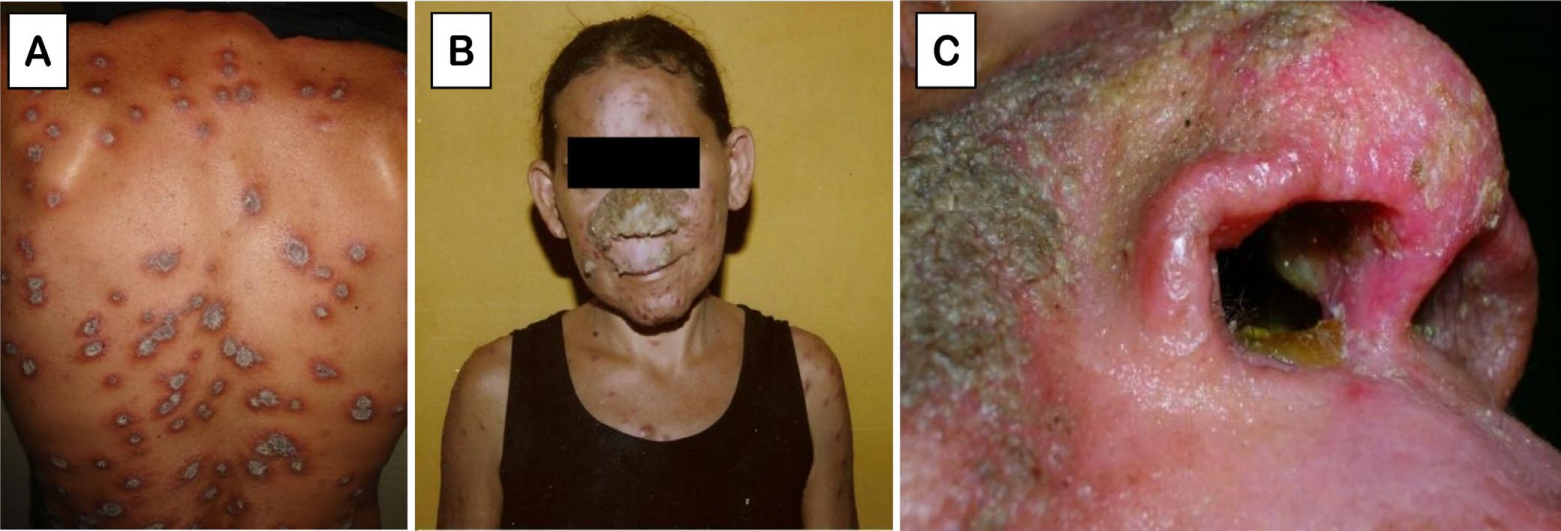
Clinical lesions at hospitalisation in November 2008, 7 months after HAART initiation. (A) Papulo‐nodular nummular plaques disseminated on the back; (B) verrucous plaque on the central face with nasal dorsum involvement and (C) close‐up view showing destruction of the nasal septum.

In November 2008, 7 months after HAART initiation and 4 months after the first signs of lesion exacerbation, the patient was hospitalised. She presented grouped papular‐erythematous lesions in the oropharynx, nodular erythematous‐infiltrated lesions on the nasal dorsum evolving into a central verrucous plaque with nasal septum destruction and erythematous‐squamous and papulo‐nodular nummular plaques disseminated on the trunk and lower limbs (Figure 1). On admission, the CD4^+^ T‐lymphocyte count had risen to 347 cells/mm^3^ and the viral load was undetectable. A skin biopsy from a back lesion showed ulcerated epidermis and lymphomononuclear infiltrate with a moderate neutrophilic component in the superficial and mid‐dermis, and direct parasitological examination of lesion scraping was positive.

Pentavalent antimonial was restarted for 30 days, while the HAART regimen was maintained. Both cutaneous and mucosal lesions fully regressed within 4 weeks after treatment restart. Over 16 years of follow‐up, no recurrence of ATL lesions was observed.

### 2.2 Immunohistochemistry

The biopsy obtained in November 2008 was processed for immunohistochemical analysis following the avidin–biotin–peroxidase complex method [15], as previously described by our group [16]. Primary antibodies, manufacturers, catalogue numbers and dilutions are detailed in Table 1. Negative controls were performed by omitting the primary antibody; healthy skin samples from individuals without dermatoses or relevant comorbidities served as baseline controls, and tissue from HIV‐negative patients with MCL caused by *Leishmania* (*Viannia*) sp. (*n* = 5) served as disease‐specific comparators. Marker expression was quantified as cell density (cells/mm^2^) from five representative photomicrographs per marker.

**TABLE 1:** Primary antibodies used for immunohistochemical analysis.

Antibody	Manufacturer	Catalogue number	Dilution
CD4	Santa Cruz Biotechnology	sc‐13573	1:50
CD8	Santa Cruz Biotechnology	sc‐1181	1:50
CD20	DakoCytomation	IS604 (clone L26)	1:50
CD68	Abcam	ab955	1:400
iNOS	Abcam	ab15323	1:200
CD163	Abcam	ab156769	1:900
IL‐10	R&D Systems	MAB217	1:700
IL‐6	Santa Cruz Biotechnology	sc‐130326	1:200
TGF‐β	Abcam	ab190503	1:100
FOXP3	Santa Cruz Biotechnology	sc‐28705	1:250
IL‐17	Santa Cruz Biotechnology	sc‐374218	1:200
IL‐1β	Elabscience	E‐AB‐40530	1:300
IL‐18	Abcam	ab68435	1:2000
NLRP3	Elabscience	E‐AB‐93112	1:600
AIM2	Elabscience	E‐AB‐10974	1:100
Caspase‐1	Elabscience	E‐AB‐40529	1:500

### 2.3 Ethical Aspects

Written informed consent for publication of clinical data and images was obtained from the patient. The study was approved by the Ethics Committee of the Tropical Medicine Department of the Federal University of Pará (CAAE 86238424.5.0000.5172; Protocol Number 7.647.541) and Medical School, São Paulo University (CAAE 86238424.5.3001.0068; Protocol Number 7.782.608).

## 3 Results

**FIGURE 2:**
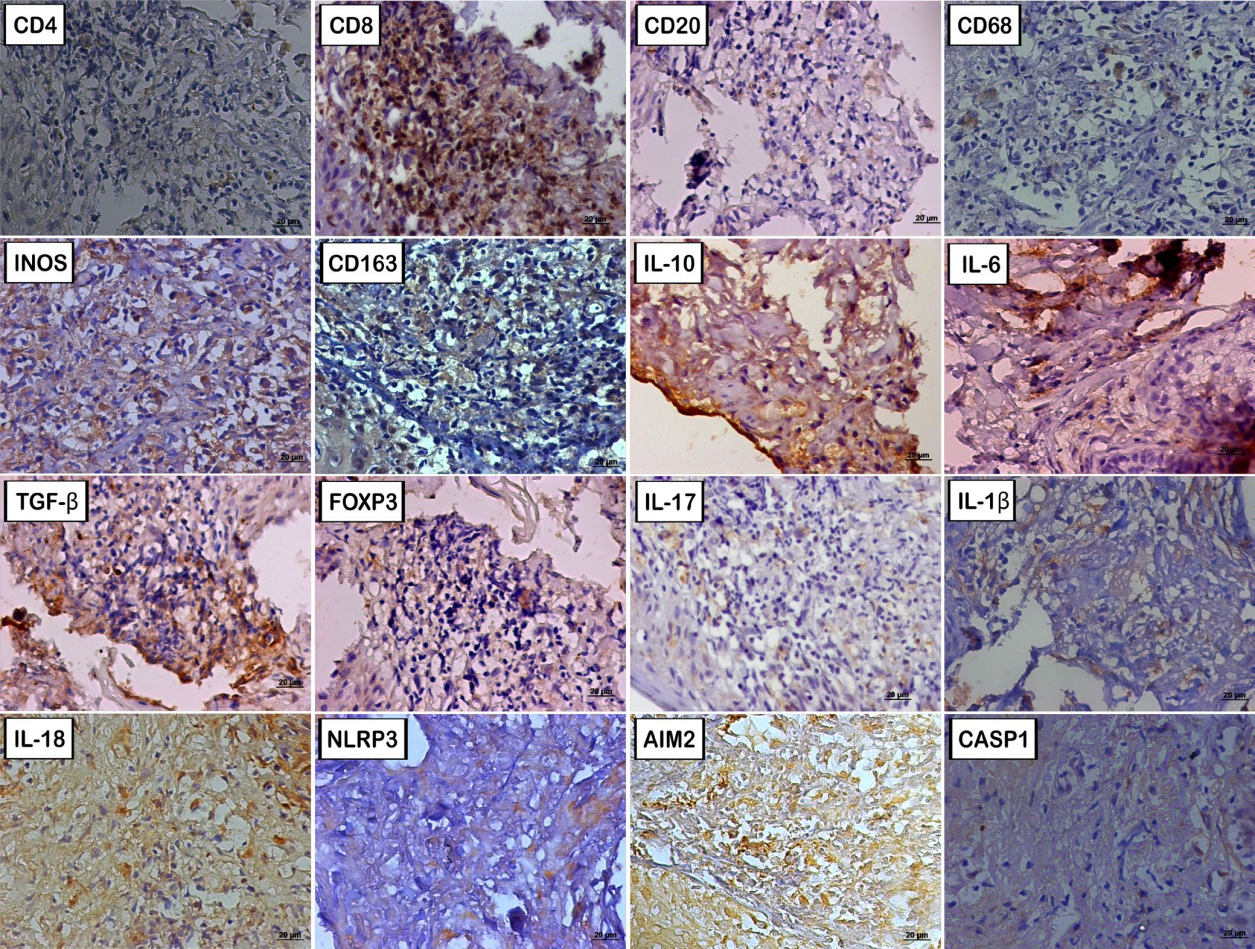
Immunohistochemical analysis of a skin biopsy from a back lesion obtained during hospitalisation in November 2008, 7 months after HAART initiation. Polymer conjugate immunohistochemical method with secondary antibodies; scale bar = 20 μm. Negative controls showed no specific labelling.

Quantitative data for all markers are summarised in Table 2 and illustrated in Figure 2. In the coinfected patient, classic markers of M1 macrophage activation (CD68, iNOS) and of Th1/Th17 profile (IL‐6, IL‐17 and IL‐1β) were substantially lower than in HIV‐negative ATL patients, while markers associated with M2 macrophage polarisation and a regulatory/Th2‐skewed profile—CD163, IL‐10, TGF‐β and IL‐18—were elevated. Among lymphocyte populations, CD4^+^, CD20^+^ and FOXP3^+^ cells were markedly reduced, whereas CD8^+^ was the most preserved subset. Inflammasome markers NLRP3, AIM2 and Caspase‐1 were also decreased.

**TABLE 2:** Cell density (cells/mm^2^) of immunohistochemical markers by study group.

Immunological marker	Coinfected patient (cells/mm^2^)	HIV‐negative ATL (cells/mm^2^)	Healthy control (cells/mm^2^)
CD4	81.40	**1700**	46.25
CD8	1212.06	**2117**	15.87
CD20	203.51	**1016**	12.60
CD68	307.54	**963**	17.80
iNOS	753.01	**808**	0.10
CD163	**352.76**	83	3.70
IL‐10	**149.25**	100	13.26
IL‐6	135.08	**300**	7.31
TGF‐β	**361.81**	317	0.11
FOXP3	226.13	**648**	3.78
IL‐17	88.19	**494**	18.64
IL‐1β	210.30	**528**	5.42
IL‐18	**497.99**	456	7.36
NLRP3	190.00	**400**	7.93
AIM2	180.00	**467**	9.42
Caspase‐1	181.00	**396**	4.52

*Note:* Values represent the mean of five representative photomicrographs per marker. HIV‐negative ATL group: patients with MCL caused by *Leishmania* (*Viannia*) sp. (*n* = 5). Healthy control group: normal skin from individuals without dermatoses or relevant comorbidities. Bold values indicate the highest cell density for each marker across the three groups.

## 4 Discussion

The diagnosis of IRIS‐associated ATL was supported by the temporal relationship between lesion exacerbation and HAART (4 months after initiation), the concomitant CD4^+^ T‐cell recovery with viral load suppression and the confirmation of active Leishmania infection. IRIS in ATL typically emerges 4–8 weeks after HAART, but onset may range from days to months, and diagnosis relies on a combination of clinical and laboratory findings [9, 11, 12, 13].

Visceral leishmaniasis/HIV coinfection has been more extensively characterised, particularly in Mediterranean countries, where IRIS‐related visceral reactivation may occur despite CD4^+^ recovery [4, 5]. Reports of ATL‐associated IRIS remain scarce and heterogeneous [9, 11, 12, 13]. Gois et al. [14] described an AIDS patient with MCL as IRIS after HAART, clinically similar to our case, reinforcing the concept that ATL reactivation under immune reconstitution may preferentially evolve towards the mucocutaneous form. Since most evidence indicates that HAART facilitates Leishmania control rather than exacerbation [10], this case underscores the paradoxical nature of IRIS. Effective antiretroviral treatment does not preclude and may even underlie this atypical inflammatory response [13].

The immunohistochemical panel was structured around four immunopathological profiles, with some markers participating in more than one, given their pleiotropic functions. The cellular/macrophage profile comprised lymphocyte populations and macrophage polarisation markers (CD4, CD8, CD20, CD68, iNOS and CD163). The regulatory profile included FOXP3, IL‐10 and TGF‐β; IL‐10 also participates in the macrophage profile as a signal of M2 alternative activation. The pro‐inflammatory profile encompassed IL‐6, IL‐17, IL‐1β and TGF‐β, the latter shared with the regulatory profile, given its dual inflammatory/immunosuppressive role. The inflammasome activation was assessed through NLRP3, AIM2, Caspase‐1, IL‐1β (shared with the pro‐inflammatory profile) and IL‐18.

The cellular/macrophage profile revealed the core disruption of this case. As expected from HIV disease stage, CD4^+^ and CD20^+^ lymphocytes were markedly reduced. Strikingly, CD8^+^ T cells were the most preserved lymphocyte subset, preserving the CD8 > CD4 hierarchy typical of *L*. (*Viannia*) sp. lesions [7]. In mucosal leishmaniasis caused by *L*. (*Viannia*) *braziliensis*, CD8^+^ lymphocytes preferentially drive tissue injury through granzyme‐mediated cytotoxicity rather than effective parasite clearance [17, 18]. In parallel, the macrophage compartment showed reduced CD68 and iNOS alongside markedly elevated CD163, indicating a shift from M1 classical to M2 alternative activation. The combination of preserved cytotoxic CD8 activity with blunted macrophage microbicidal capacity provides a coherent explanation for the tissue destruction observed despite parasite persistence.

The regulatory and pro‐inflammatory profiles reinforced this interpretation. Elevated IL‐10 and TGF‐β, with reduced FOXP3, argue against a Treg‐dominant mechanism and suggest these cytokines originate predominantly from the M2‐polarised macrophage population [7], consistent with CD163 elevation. The pro‐inflammatory profile showed reduced IL‐6, IL‐17 and IL‐1β, indicating a weakened Th1/Th17 axis, divergent from the classical response required to control *L*. (*Viannia*) sp., in which IFN‐γ‐producing cells normally predominate [7, 8]. Together, the reduced effector cytokines and expanded regulatory/M2 mediators reconfigure the tissue milieu towards a Th2‐skewed, parasite‐permissive pattern.

The inflammasome profile showed a dissociated pattern. NLRP3, AIM2 and Caspase‐1 were reduced, suggesting that canonical inflammasome pathways were not adequately engaged against the parasite. Since inflammasome activation contributes to parasite restriction in the more resistant ATL forms [19], its blunting is consistent with the persistence of amastigotes in lesion scrapings. IL‐18, however, was isolatedly elevated. This is a relevant finding since IL‐18 is most expressed in anergic diffuse forms caused by *L*. (*L*.) *amazonensis*, which also follow a Th2‐skewed pattern [8, 19]. This dissociation aligns our patient's profile with Th2‐polarised, susceptible ATL phenotypes.

Treatment options for ATL‐IRIS include pentavalent antimonials or amphotericin B, occasionally combined with corticosteroids [2, 9, 11, 12, 20]. Couppié et al. [3] reported higher recurrence and lower cure rates in HIV‐positive patients compared to non‐HIV controls. In the present case, no recurrence was observed over 16 years, an outcome that may reflect therapy adherence, sustained HAART and species‐related factors.

## 5 Conclusion

This case documents a paradoxical IRIS manifestation in ATL‐HIV coinfection characterised by three converging immunopathological findings: (i) a Th2/M2‐polarised tissue milieu with suppression of Th1/Th17 responses; (ii) partial preservation of the CD8+ compartment in a context of blunted macrophage microbicidal activity, plausibly contributing to tissue destruction through cytotoxic mechanisms; and (iii) a dissociated inflammasome profile with reduced expression of NLRP3, AIM2, IL‐1β and isolated IL‐18 elevation. Despite initial severity, pentavalent antimonial combined with sustained HAART led to complete resolution without recurrence over 16 years. The case reinforces the need for early recognition of IRIS in coinfected patients from endemic areas and invites further investigation into the role of CD8‐mediated mechanisms in ATL‐HIV tissue injury.

## Author Contributions

**Marilia Brasil Xavier:** conceptualisation, investigation, methodology, project administration, visualisation and writing – review and editing. **Claudia Maria de Castro Gomes:** resources, validation and writing – original draft. **Rita Catarina Medeiros Sousa:** investigation. **Edna Aoba Yassui Ishikawa:** investigation and resources. **Elza Baía de Brito:** investigation and resources. **Silvia Ferreira Rodrigues Müller:** investigation. **Lucas dos Santos Fontes:** formal analysis and writing – original draft. **João Augusto Gomes de Souza Monteiro de Brito:** investigation. **Larissa dos Santos Alcantara:** investigation. **Carlos Eduardo Pereira Corbett:** funding acquisition, resources, supervision and writing – review and editing.

## Funding

The research was supported by São Paulo Research Foundation (FAPESP), grant #2014/50315‐0. This study was financed in part by the Coordenação de Aperfeiçoamento de Pessoal de Nível Superior – Brasil (CAPES) – Finance Code 001.

## Conflicts of Interest

The authors declare no conflicts of interest.

## Data Availability

The data that support the findings of this study are available on request from the corresponding author. The data are not publicly available due to privacy or ethical restrictions.
